# The impact of climate change on depression in rural Chinese older adult

**DOI:** 10.3389/fpubh.2025.1610597

**Published:** 2025-06-04

**Authors:** Chen Liu, Kaihua Zhang, Chenze Zhao, Yufeng Yan, Jinye Li

**Affiliations:** School of Business Administration, Zhongnan University of Economics and Law, Wuhan, Hubei, China

**Keywords:** climate change, rural older adult, depression, low temperature, mediating mechanisms

## Abstract

**Introduction:**

In recent years, the impact of climate change on the economy and society has become increasingly significant, with depression emerging as a major factor hindering individuals' daily functioning and quality of life. Rural older adult, due to their low income and inadequate social security, face particularly prominent depressive symptoms. However, existing research has predominantly focused on developed countries, with insufficient attention paid to depressive disorders among rural older adult populations in China.

**Methods:**

This study, based on data from the China Health and Retirement Longitudinal Study (CHARLS) from 2013 to 2020 and meteorological monitoring data, employs a two-way fixed effects model to examine the effects of climate change on depressive symptoms in rural older adult.

**Results:**

The findings reveal that: (1) extreme low temperatures are the primary climatic factor increasing depression risks of rural older adult; (2) the depression of women, those with low education levels, those engaged in agricultural activities, and widowed individuals is more significantly affected by low temperatures; (3) climate change directly heightens depression risks among rural older adult through heightened social isolation and loneliness. (4) climate change indirectly exacerbates depression risks through deteriorating physical health, reduced outdoor activities, declining cognitive abilities, and decreased sleep quality.

**Discussion:**

This study provides empirical evidence for policymakers to assess the health costs of climate change and propose targeted interventions for depressive disorders.

## 1 Introduction

In recent years, the phenomenon of climate change has become more pronounced globally. The trend of global warming and the heightened frequency of extreme weather events have adversely affected macroeconomic dimensions, including agricultural productivity and industrial output. Extreme weather elevates the probability of farmers falling into extreme poverty, with a U-shaped relationship observed between standardized annual mean temperature and relative poverty. Under optimal climatic conditions, the likelihood of farmers falling into relative poverty is minimized (1). However, the impact of climate change on agriculture is not unilaterally negative but exhibits complex trade-offs between beneficial and detrimental effects. Through technological advancements and market mechanisms, global warming may yield net economic benefits for U.S. agriculture in the long term (2). This apparent paradox underscores the complexity of climatic influences on agriculture: moderate temperature increases over extended periods may enhance output by prolonging crop growth cycles, yet extreme weather events persistently pose direct threats through seasonal disruptions. Such dual effects have been robustly validated in cross-national studies of India and the United States, where natural disasters, including extreme weather, drive labor migration from agriculture to secondary and tertiary sectors such as construction, triggering inter-sectoral employment reallocation (3).

Furthermore, studies on Chinese industrial enterprises reveal that temperature fluctuations significantly reduce total factor productivity (TFP), with heatwaves exhibiting particularly pronounced adverse effects (4, 5). The inhibitory impact of high temperatures on industrial production demonstrates remarkable consistency across diverse climatic zones. Research on 28 Caribbean nations confirms that rising temperatures substantially suppress industrial output, with the industrial sector experiencing greater vulnerability compared to agriculture (6). Mechanistic analyses indicate that heat-induced declines in worker attendance and productivity systematically diminish industrial yields (7).

At the micro level, climate change detrimentally affects human health and household income. U.S.-based studies demonstrate that community healthcare services reduce heat-related mortality by ~14.2%, though no significant mitigation is observed for cold-related mortality (8). Temperature effects exhibit life-course plasticity among vulnerable populations, prompting heightened research focus on the thermal sensitivity of pregnant women and fetuses. Analysis of Indian survey data reveals that prenatal heat exposure elevates infant mortality (9), a finding corroborated in China, where prenatal extreme weather events significantly reduce neonatal birth weight (10). Notably, temperature-induced health sequelae extend beyond early life stages. Chinese data indicate that both extreme heat and cold substantially increase the incidence and mortality of chronic conditions such as cardiovascular and cerebrovascular diseases (11). Collectively, these findings construct an intergenerational health impact chain of climate change: from fetal developmental impairments to accelerated onset of chronic diseases in adulthood, culminating in complex socioeconomic burdens. In developed economies, analysis of 40-year U.S. county-level data demonstrates temperature-income correlations: per capita expenditures increase by $20 when temperatures exceed 30°C (12). As a result, high temperatures push up household expenditures, revealing that warming erodes the momentum of economic development by reducing labor productivity and increasing the cost of living, confirming the urgency of climate adaptation investments.

According to the World Health Organization (WHO) definition in 2017, mental health refers to a state of continuous, positive, and effective psychological wellbeing. Notably, climate change exerts non-negligible impacts on depression. Analysis of U.S. emergency department data reveals that extreme weather events substantially elevate the incidence of psychiatric disorders and violence-related injuries (13). This pattern is further corroborated by global evidence—a synthesis of 17 studies spanning the United States, Turkey, Japan, South Korea, and other regions demonstrates that climatic stressors (e.g., low winter temperatures, extreme thermal variations, or heatwaves in high-altitude zones) elevate depression and suicide risks, mediated by hypoxia-induced metabolic strain and neurotransmitter dysregulation (14). These empirical findings align with theoretical frameworks explaining climate-psychopathology cascading effects. Further mechanistic modeling identifies that climate change impacts depression not only through direct physiological stressors that impair individual wellbeing but also via compounded effects from eroded social cohesion and diminished adaptive capacities (15).

To systematize these multidimensional interactions, a tripartite taxonomy of depression impacts has been established: direct effects (e.g., trauma from extreme weather), indirect effects (e.g., eco-anxiety regarding future risks), and psychosocial effects (e.g., community disintegration post-migration or drought) (16). Crucially, this analytical framework integrates acute clinical observations with systemic analyses, demonstrating how transient temperature shocks evolve into chronic depression burdens through cumulative socioeconomic vulnerabilities.

Concurrently, global aging trends demand urgent attention, with China experiencing the world's most rapid growth in older adult populations. According to China's National Bureau of Statistics, by the end of 2022, individuals aged 60 and above will reach 280 million, constituting 19.8% of the total population (17). Rural Chinese older adult are characterized by large population size, fragile pension systems, and low health literacy. Compared to urban counterparts, rural elders face unique challenges, including limited access to healthcare, social support, and technological infrastructure.

The “China National Mental Health Development Report (2021–2022)” indicates that the depression detection rate among those aged 55 and above is higher than that of the 45–54 and 35–44 age groups, with urban residents having a lower depression detection rate than rural residents—a disparity potentially attributable to rural-urban divides in healthcare accessibility, socioeconomic status, and depression stigma (18). Specifically, rural communities confront multifaceted barriers, including inadequate mental health infrastructure, economic constraints on healthcare-seeking, and cultural taboos against help-seeking behaviors. Their unique vulnerabilities—such as social isolation, loneliness, and restricted medical access (19–22) —profoundly affect emotional, physical, and cognitive health (23–25). China's rapid aging and urbanization have precipitated large-scale migration of younger generations from rural to urban areas, leaving many older adult without familial care and exacerbating their challenges (26). The absence of proximate kin may intensify feelings of loneliness and hopelessness, elevating depression risks (27, 28). For instance, a rural Chinese study found depression symptom prevalence of 44.2% among empty-nest older adult, significantly higher than the 26.3% observed in non-empty-nest counterparts (29, 30). Furthermore, insufficient mental stimulation and social interaction accelerate cognitive decline, impairing memory, attention, and executive function, thereby increasing dementia risks (31, 32).

Rapid industrialization has accelerated greenhouse gas emissions, intensified extreme weather events, and disrupted ecosystems, thereby amplifying global climate risks. This industrial growth exacerbates socioeconomic vulnerabilities, particularly in regions with inadequate infrastructure, widening climate-induced health and economic disparities among marginalized groups such as rural older adult. Consequently, these structural inequities, compounded by age-specific vulnerabilities, render rural older adult depression an urgent policy priority.

Despite growing health awareness, critical research gaps persist. First, existing climate change studies predominantly focus on developed nations, neglecting developing countries characterized by fragile industrial bases, environmentally neglectful high-input/high-emission models, and acute climate challenges. Second, within developing contexts, stigma and limited depression literacy exacerbate psychological issues, yet research targeting these populations remains sparse.

Collectively, prior studies provide a robust theoretical foundation. Investigating mechanisms linking climate change to rural older adult depression carries urgent practical significance. This study addresses these gaps by analyzing climate change and depression data from Chinese rural older adult, incorporating longitudinal tracking data (48 months) from the CHARLS database (2013, 2015, 2018, 2020). It delineates regional and temporal temperature variation patterns across China and corresponding mental health statuses among heterogeneous rural older adult subgroups. Theoretical analysis explores potential climate impacts and mediating pathways, followed by empirical evaluation using Stata and two-way fixed-effects models to assess climate-depression associations. The study compares effect magnitudes, directional trends, and heterogeneities, concluding with evidence-based policy recommendations.

Focusing on China, this research fills critical voids in existing literature dominated by developed-country contexts and neglect of rural older adult depression. By elucidating climate change impacts and pathways on this vulnerable demographic, it aids governments and communities in comprehending climate-related health costs and formulating mitigation/adaptation strategies. The findings also offer valuable insights for other nations confronting similar challenges.

## 2 Theoretical mechanism

### 2.1 Direct impact of climate change on rural older adult depression

#### 2.1.1 Potential pathways through social isolation and loneliness

Climate change encompasses a range of natural and human-induced changes in the Earth's climate system. Changes in the Earth's average surface temperature are the most direct and visible manifestation of climate change. Global warming has accelerated over the past few decades as a result of increased emissions of greenhouse gases. There has been an increase in the frequency and intensity of extreme weather events, including heat waves, droughts, heavy rains, floods, hurricanes and typhoons.

Existing literature suggests that climate change may influence depression outcomes through psychosocial pathways such as social isolation and loneliness, though these specific mechanisms were not empirically tested in the current study. Facing the uncertainties and threats of natural disasters brought by climate change can worsen the living conditions of rural older adult, leading to anxiety, depression, and other mental health issues (33).

Additionally, the most direct feature of climate change is the unusual fluctuations in temperature, which can lead to extreme weather events. These events affect agricultural production, increase the frequency and intensity of extreme weather, and exacerbate the unpredictability of seasonal changes, thereby increasing the uncertainty in the lives of the older adult (34). Moreover, abnormal temperatures significantly impact agricultural production, threatening the economic security of rural older adult and increasing concerns about food safety. This reduces their economic resilience and sense of control over their lives, perpetuating a vicious cycle among rural older adults, where chronic powerlessness fosters loss-of-control anxiety, linking economic vulnerability to social withdrawal and self-worth erosion, thereby significantly elevating depression risks. increasing the risk of isolation and potentially leading to anxiety, depression, and other mental health issues (35).

Climate change exacerbates the environmental pressures and uncertainties faced by rural older adult. The Health Belief Model, a theoretical framework used to explain and predict individual health behaviors (36), suggests that if the older adult perceive climate change as a threat to their health and feel powerless to change the environment or lack coping resources, it may lead to anxiety, depression, and other psychological issues. Furthermore, as the impact of climate change on life gradually intensifies, prolonged exposure may lead to more severe mental health consequences, known as “climate anxiety” (defined as persistent worry, fear, and emotional responses to the perceived or experienced threats of climate change and its environmental consequences) or “eco-anxiety” (defined as anxiety or worry they felt about climate change and its effects) (37).

Temperature fluctuations, by altering rainfall patterns and increasing temperatures, adversely affect agricultural production. These environmental changes may force rural older adult to alter their lifestyles, such as alterations in crop cultivation patterns and diminished water resource access, may escalate psychosocial stress and amplify perceived life uncertainties, fostering fear and helplessness among affected populations (38).

This contextual interpretation suggests that climate-related social isolation mechanisms warrant dedicated investigation in future research. The current study's identification of low-temperature impacts on depression outcomes provides empirical grounding for subsequent exploration of these psychosocial pathways through targeted mixed-methods approaches.

#### 2.1.2 The composite impact of structural shocks on depression

Climate change triggers multiple systemic risks by disrupting both the physical environment and social structures. First, housing loss: Extreme weather events directly destroy the housing of rural older adult populations, leading to deteriorating living conditions or homelessness, which exacerbates insecurity and increases the risk of post-traumatic stress disorder (PTSD) (39). Second, breakdown of family systems: Climate disasters may cause family casualties or forced displacement, weakening family support networks. Widowed older adult individuals, in particular, face heightened isolation due to the absence of spouses (40). Third, erosion of community support: Climate change undermines rural economic foundations, reducing community resources and mutual aid capacity. This further restricts older adult populations' access to healthcare and social services (41). These structural changes and climate pressures form a vicious cycle—economic vulnerability limits individual adaptive capacity, while fractured social bonds weaken collective resilience, ultimately amplifying depression risks.

### 2.2 Indirect impact of climate change on rural older adult depression

#### 2.2.1 Physical health

Climate change affects depression of the older adult by impacting their physical health, represented by the pathway “climate change → physical health → depression”. The frequent occurrence of extreme high and low temperatures is particularly dangerous for the older adult, as physical ailments in rural older adult exacerbate mental distress through socioeconomic strain (e.g., healthcare costs deepening poverty), limited medical access prolonging untreated conditions, and eroded social networks compounding isolation—collectively fueling anxiety, depression, and perceived helplessness in aging populations. High temperatures can increase the risk of heatstroke, dehydration, and cardiovascular diseases, while extreme low temperatures can increase the burden on the heart, causing or exacerbating respiratory and circulatory system problems (42). Temperature changes can also affect the geographical distribution of many infectious diseases, increasing the risk of health problems, including mosquito-borne diseases, altered patterns of infectious disease transmission, foodborne illnesses, and heat-related diseases (43).

According to Maslow's hierarchy of needs, human needs can be divided into five levels, from basic physiological needs to self-actualization (44). For rural older adult, deteriorating physical health can affect their social participation, making it difficult for them to pursue personal interests, hobbies, or goals. Glass et al. pointed out that the lower the level of social integration among the older adult, the more likely they are to experience loneliness and greater psychological stress (45). Therefore, preventing depression in the older adult and maintaining good physical health are crucial. This not only relates to the quality of life of the older adult but also helps them feel more satisfied and happy psychologically.

Hypothesis 1. Climate change affects the depression of rural older adult by impacting their physical health.

#### 2.2.2 Frequency of outdoor activities

In addition to directly affecting physical health, climate change can also impact the frequency of outdoor activities among rural older adult, thereby affecting their depression. This pathway is represented as “climate change → frequency of outdoor activities → depression.” The mechanism through which climate change affects the depression of rural older adult may lie in its ability to limit their capacity and willingness to engage in outdoor activities. A lack of outdoor activities can reduce social opportunities and interactions with the natural environment, negatively impacting their mental health.

Climate change can also worsen air quality, exacerbating respiratory and cardiovascular problems, which is particularly harmful to older adult individuals with chronic diseases, leading them to avoid outdoor exercise and activities (46). Regular outdoor activities can provide natural healing (defined as natural environments enhance specific groups' physical and mental health through physical regulation and behavioral promotion), directly enhancing the psychological wellbeing of the older adult. Contact with nature is believed to improve mood, reduce stress, and even increase life satisfaction (47). Regular participation in outdoor activities, such as walking, gardening, or cycling, can improve cardiovascular health, increase muscle strength and flexibility, and lead to better self-perception and quality of life, thereby indirectly improving mental health (48).

Hypothesis 2. Climate change affects the depression of rural older adult by impacting the frequency of outdoor activities.

#### 2.2.3 Cognitive ability

Climate change can also affect cognitive abilities, thereby impacting the depression of rural older adult. This pathway is represented as “climate change → cognitive ability → depression”. The mechanism through which climate change affects rural older adult may lie in its ability to accelerate cognitive decline through various mechanisms, thereby affecting their depression.

From a physiological perspective, extreme weather events caused by climate change negatively impact cognitive abilities, including attention, memory, and decision-making (49). According to environmental stress theory, natural disasters and changes in human lifestyles caused by climate change can lead to chronic stress, which is believed to reduce cognitive abilities by affecting the hippocampus and prefrontal cortex of the brain (50). Low temperatures place additional burdens on the heart and circulatory systems of the older adult, potentially slowing blood circulation and reducing blood supply to the brain. As the brain is the central control for cognitive functions, reduced blood supply can adversely affect cognitive abilities (51). Malnutrition is significantly associated with cognitive function, with greater severity of malnutrition linked to poorer cognitive performance. This association is particularly pronounced among illiterate women aged 90 and above (52). In rural areas, the impact of climate change on food production and water availability indirectly affects people's nutritional status and health. Particularly in developing and low-income countries, malnutrition can affect long-term cognitive development.

The stress-vulnerability model explains that the intrinsic vulnerability due to declining cognitive abilities, combined with external pressures from daily life challenges, can jointly lead to the emergence of depression issues (53).

Hypothesis 3. Climate change affects the depression of rural older adult by impacting their cognitive abilities.

#### 2.2.4 Sleep duration and quality

Climate change, especially extreme temperatures, can lead to depression by impacting sleep duration and quality. This mechanism is represented as “climate change → sleep quality → depression”. The mechanism through which climate change affects rural older adult may lie in its ability to disrupt normal sleep patterns, affecting physiological functions and thereby impacting mood and mental health. An appropriate sleep environment temperature is crucial for ensuring sleep quality, and extreme climatic conditions can disrupt this balance, leading to sleep disorders. There is a close relationship between sleep quality and depression.

Extreme temperatures can disrupt sleep balance, affecting overall sleep quality. At an appropriate bedroom temperature, the body is more likely to enter sleep, while temperatures that are too high or too low can interfere with the normal sleep cycle, including rapid eye movement (REM) and non-rapid eye movement (NREM) sleep. The alternation between REM and NREM sleep is key to high-quality sleep, and unsuitable temperature environments can disrupt these stages, causing sleep disorders and reducing sleep duration and quality (54).Environmental changes caused by climate change, such as significant indoor-outdoor temperature differences and humidity changes, pose challenges to maintaining a good sleep environment, thereby affecting sleep quality. Raymann and Van Someren found that an appropriate sleep environment temperature is crucial for ensuring sleep quality, and extreme climatic conditions can disrupt this balance, leading to sleep disorders (55).

Sleep deprivation or sleep disorders have been linked to many depression issues, including anxiety, depression, and more severe emotional and behavioral problems. According to stress theory, temperature changes, as a long-term external stressor caused by climate change, can increase individual stress levels by reducing sleep quality. This long-term stress state not only affects individual mental health but can also lead to the development of psychological disorders (56).

Hypothesis 4. Climate change affects the depression of rural older adult by impacting sleep duration and quality.

## 3 Methodology

### 3.1 Dataset

This study uses a two-way fixed effects model to analyze the causal relationship between climate change and the depression of rural older adult in China and to explore the mechanisms. Based on 4 years of CHARLS data, the study constructs depression indicators based on residents' reported metrics and combines them with meteorological data from monitoring stations across China to conduct empirical research. Heterogeneity analysis is conducted based on gender, age, whether they have pension insurance, whether they have medical insurance, and parents' education levels to examine the differential impacts of climate change on the depression of different groups. Finally, the study empirically tests whether the mechanisms proposed in the theoretical analysis hold.

### 3.2 Variables

#### 3.2.1 Explained variable

**Figure 1:**
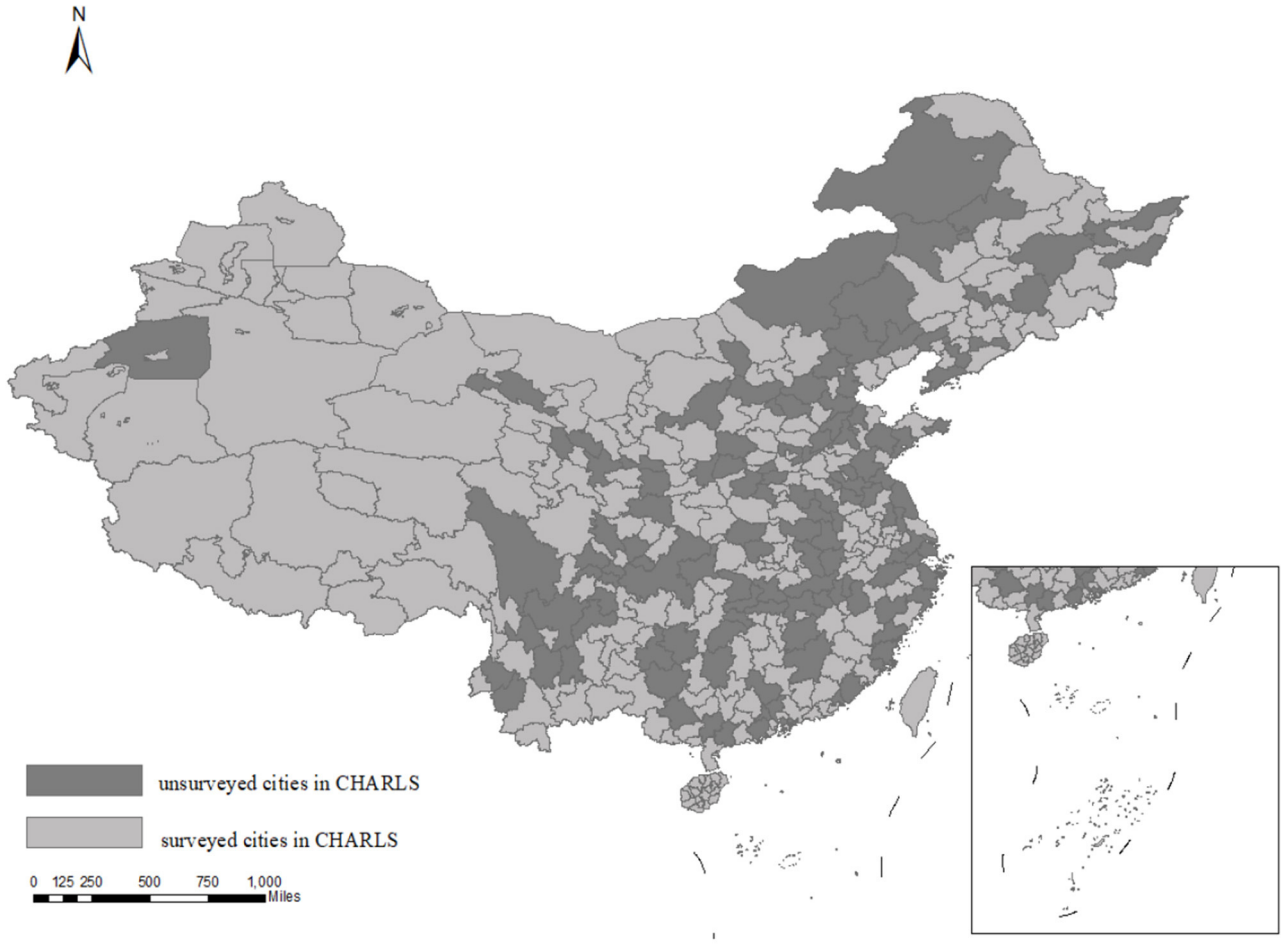
Distribution of CHARLS survey scope. This map is based on the standard map [GS (2023) No. 2764] from the Standard Map Service website of the Ministry of Natural Resources.

The explained variable is the depression of older adults. In line with international standards, individuals aged 65 and above are defined as older adults. Depression data are sourced from the China Health and Retirement Longitudinal Study (CHARLS), a nationally representative micro-level dataset of households and individuals aged 45 and above in China, conducted by Peking University. The geographic distribution of the survey is illustrated in Figure 1. This study utilizes rural older adult data from the 2013, 2015, 2018, and 2020 CHARLS waves, yielding 18,668 valid observations.

Due to data limitations, we are unable to assess the psychological activities of individual respondents. Therefore, this study focuses on the depression status of rural older adults. The CHARLS survey includes sections on depression and cognitive ability to evaluate depression, utilizing the 10-item Center for Epidemiological Studies Depression Scale (CES-D). CES-D scores were reverse-coded, with higher scores indicating more severe depressive symptoms. This scale measures multidimensional emotional states over a short period and is widely used to assess depressive symptoms, with strong psycho-metric reliability and validity. The 10 items in the CES-D scale are as follows:

1. I was bothered by small things.
2. I had difficulty concentrating.
3. I felt depressed.
4. I felt that everything I did was an effort.
5. I felt hopeless about the future.
6. I felt fearful.
7. My sleep was restless.
8. I felt unhappy.
9. I felt lonely.
10. I felt that I could not continue with my life.

Respondents were asked to rate each item on a 4-point scale (excluding “don't know” and “refuse to answer”): most of the time (1 point), some or a little of the time (0.5 points), rarely (0.5 points), and not at all (0 points). Higher CES-D scores indicate poorer mental health and more pronounced depressive symptoms.

#### 3.2.2 Core explanatory variable

The core explanatory variable is climate change. Given its multidimensional and complex nature, this study uses temperature variation as the primary proxy. Specifically, a series of dummy variables representing different average temperature intervals are constructed: ≥30°C, 25–30°C, 20–25°C, 15–20°C, 10–15°C, 5–10°C, and <5°C. Given the temporal alignment between depression measurement and weather exposure windows, this study links meteorological data to survey timelines with precision: while the CES-D scale evaluates depressive symptoms over the preceding week, other survey items capture depression status across the past month. To reconcile these timeframes and establish temporal precedence (i.e., weather preceding outcomes), we operationalize weather exposure as the 30-day period before the survey date, thereby isolating how prior climatic conditions may prospectively influence depression while mitigating reverse causality risks.

#### 3.2.3 Control variables

On one hand, this study controls for weather variables that may correlate with temperature and affect mood: sunshine duration, precipitation, relative humidity, and average wind speed. Meteorological data are sourced from the China Meteorological Data Sharing Service System (CMDSSS). Using the location data of respondents from the 2013 CHARLS survey, this study matches city-level meteorological data with individual responses to examine the link between climate change and depression. The Inverse Distance Weighting (IDW) method is applied to calculate the weighted aver-age of daily meteorological variables for each city from 2010 to 2015.

On the other hand, individual characteristics that may influence depression are also controlled for: whether the individual has pension insurance and whether the in-dividual has health insurance. Due to significant missing data on household income, these variables serve as proxies for economic status and future living standards.

In summary, this study utilizes four waves of CHARLS data (2013, 2015, 2018, and 2020), covering 48 months of longitudinal data. After excluding samples with severe missing data on CES-D scores, individual characteristics, and weather variables, the final dataset includes 18,668 rural older adults from 95 cities.

Table 1 displays the descriptive statistics of variables.

**Table 1:** Descriptive statistics of variables.

**Variable name**	**Observations**	**Mean**	**Std. dev**.	**Min**	**Max**
**Depression indicators:**					
CES-D Score	18,668	2.807	1.513	0	6
Bothered by small things	18,668	0.300	0.350	0	1
Difficulty concentrating	18,668	0.290	0.350	0	1
Felt depressed	18,668	0.290	0.340	0	1
Everything felt like an effort	18,668	0.300	0.370	0	1
Felt hopeless	18,668	0.4707	0.420	0	1
Felt fearful	18,668	0.120	0.260	0	1
Restless sleep	18,668	0.350	0.390	0	1
Felt unhappy	18,668	0.389	0.390	0	1
Felt lonely	18,668	0.180	0.320	0	1
Could not continue with life	18,668	0.120	0.270	0	1
**Temperature variables:**					
Monthly average temp. <5°C	18,668	0	0.0200	0	1
Monthly average temp. [5°C−10°C)	18,668	0	0.0300	0	1
Monthly average temp. [10°C−15°C)	18,668	0.0100	0.0900	0	1
Monthly average temp. [15°C−20°C)	18,668	0.0500	0.210	0	1
Monthly average temp. [20°C−25°C)	18,668	0.260	0.440	0	1
Monthly average temp. [25°C−30°C)	18,668	0.650	0.480	0	1
Monthly average temp. ≥30°C	18,668	0.0400	0.190	0	1
Sunshine duration (hours)	18,668	193.0	50	27.93	359.9
Precipitation (mm)	18,668	176.4	106.1	0.210	745.8
Relative humidity (%)	18,668	77.03	7.170	41.69	92.89
Average wind speed (m/s)	18,668	4.800	1	2.660	8.640
Has pension insurance	18,668	0.320	0.210	0	1
Has health insurance	18,668	0.470	0.092	0	1

### 3.3 Methods

This study uses a two-way fixed effects model to analyze the causal relationship between climate change and the depression of rural older adult in China and to explore the mechanisms. Based on 4 years of CHARLS data, the study constructs depression indicators based on residents' reported metrics and combines them with meteorological data from monitoring stations across China to conduct empirical research. Heterogeneity analysis is conducted based on gender, age, whether they have pension insurance, whether they have medical insurance, and parents' education levels to examine the differential impacts of climate change on the depression of different groups. Finally, the study empirically tests whether the mechanisms proposed in the theoretical analysis hold.

Based on the preceding analysis, this study employs a two-way fixed effects model as specified in Equation 1 to empirically evaluate the relationship between climate change and the depression of rural older adult:


(1) $$\begin{array}{l} Y_{\mathrm{icym}}=\beta_{0}+\sum_{n\mathrm{= 1}}^{7}\beta_{n}\times {\mathrm{MeanTemp}}_{\mathrm{cm}}+X_{\mathrm{icy}}\theta ++\mu_{i}\\ \;+\delta_{c}+\pi_{y}+\omega_{m}+\varepsilon_{□} \end{array}$$

In Equation 1, **Y**_**icym**_ denotes the depression problem index of individual **i** in city **c**, year **y**, and month **m**. **MeanTemp**_**cym**_ is a set of binary variables indicating whether the monthly average temperature in city **c** and month **m** falls within a specific interval. This study categorizes monthly average temperatures into seven intervals: <5°C, 5–10°C, 10–15°C, 15–20°C, 20–25°C, 25–30°C, and ≥30°C. Following Fanger (1970), who identified 22–23°C as the neutral temperature range (57), the 20–25°C interval is selected as the reference group to avoid multicollinearity. Consequently, the coefficients of interest **(β**_**1**_, ***β***_**2**_, ***β***_**3**_, ***β***_**4**_, ***β***_**6**_, ***β***_**7**_**)** reflect the differential effects of each temperature interval on rural older adults' depression relative to the reference group (20–25°C).

**X**_**iy**_ represents individual-level control variables and city-level weather control variables. **μ**_**i**_ and **δ**_**c**_ capture individual and city fixed effects, respectively. **π**_**y**_ denotes year and month fixed effects. **ε** represents the error term clustered at the city level. Prior to regression analyses, all control variables were regressed out to eliminate the effects of potential confounders. As a result, the final model only reports estimates of the core explanatory variables. Fixed effects (individual, city, year, month) are parametrically estimated by introducing dummy variables grouped together with coefficients that are not presented in the table but are fully controlled for.

This study further constructs a mediation effect model to empirically test the specific pathways through which climate change affects the depression of rural older adult. The stepwise method is used to test the mediating effects of physical health, outdoor activity frequency, cognitive ability, and sleep duration on depression. The regression models are specified as follows:


(2) $$\begin{array}{l} Y_{\mathrm{icym}}=\beta_{0}+\overset{7}{\underset{n\mathrm{= 1}}{\sum }}\alpha_{n}\times {\mathrm{MeanTemp}}_{\mathrm{cm}}+X_{\mathrm{icy}}\theta +\mu_{i}\\ \;+\delta_{c}+\pi_{y}+\omega_{m}+\varepsilon_{□} \end{array}$$

(3) $$\begin{array}{l} {\mathrm{MED}}_{\mathrm{icym}}=\beta_{0}+\overset{7}{\underset{n\mathrm{= 1}}{\sum }}\beta_{n}\times {\mathrm{MeanTemp}}_{\mathrm{cm}}+X_{\mathrm{icy}}\theta +\mu_{i}\\ \;+\delta_{c}+\pi_{y}+\omega_{m}+\varepsilon_{□} \end{array}$$

(4) $$\begin{array}{l} Y_{\mathrm{icym}}=\beta_{0}+\overset{7}{\underset{n\mathrm{= 1}}{\sum }}\gamma_{n}\times {\mathrm{MeanTemp}}_{\mathrm{cm}}+{\rho \text{MED}}_{\mathrm{iy}}\\ \;+X_{\mathrm{icy}}\theta +\mu_{i}+\delta_{c}+\pi_{y}+\omega_{m}+\varepsilon_{□} \end{array}$$

Where **MED**_**iy**_ is the mediating variable, indicating the pathway through which climate change affects physical health. Equations 2–4 represent the mediation effect testing procedure. First, the coefficient **α** is tested, followed by the coefficients ***β*** and **γ**. If both are significant, the mediation effect is significant. Additionally, **α** represents the total effect of climate change on the depression of rural older adult; ***β*** represents the effect of climate change on the mediating variable; **γ** represents the direct effect of climate change on the depression of rural older adult after controlling for the mediating variable; and **ρ** represents the effect of the mediating variable on the depression of rural older adult after controlling for climate change. The total effect equals the direct effect plus the mediation effect, i.e., γ+β^*^ρ. Since the baseline model regression satisfies the condition that **α** is significant (as shown in Column (4) of Table 4), this article directly tests the mediation effect using Equations 3, 4.

## 4 Characteristics of climate change and depression of rural older adult

### 4.1 Characteristics of climate change

#### 4.1.1 Temporal trends of different climate types in China

**Figure 2:**
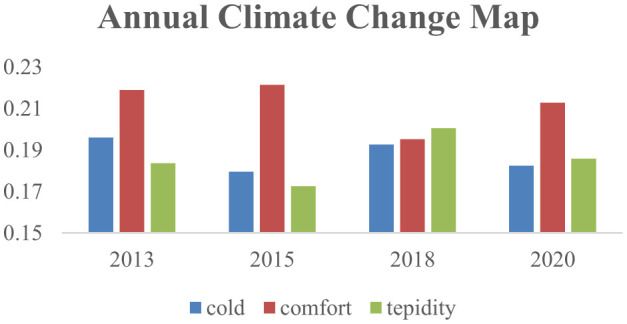
Frequency of temperature occurrences of various types in China by year. Data source: China meteorological data sharing service system.

Figure 2 shows the frequency of different temperature types in China over the years, based on data from the China Meteorological Data Sharing Service System. The frequency of warm climates shows a wavy trend but generally increases, peaking in 2018, with 2020′s frequency higher than in 2013 and 2015. Horizontally, the most common temperature type across all years is comfortable climate, except in 2018 when warm climates were most frequent, and cold climates were also relatively high. In 2015, the climate was most suitable, with the highest frequency of comfortable cli-mates and lower frequencies of cold and warm climates.

#### 4.1.2 Time variation trends of different types of climate in different regions of China

Table 2 shows that in the eastern region, warm climates have the highest frequency of occurrence, followed by comfortable and cold climates. In the central region, the climate frequency is more inclined toward warm climates. In the western region, the most frequent occurrence is cold weather, and it is also the most frequent occurrence of cold climate in various regions.

**Table 2:** Frequency of temperature appearance by region, year, and type.

**Eastern, Central, and Western Regions**	**Temperature type**	**2013**	**2015**	**2018**	**2020**
East	Cold	0.0459	0.0439	0.0439	0.0383
	Comfort	0.0707	0.0741	0.0662	0.0750
	Tepidity	0.0935	0.0941	0.1023	0.0973
Central	Cold	0.0653	0.0518	0.0637	0.0552
	Comfort	0.0662	0.0709	0.0543	0.0592
	Tepidity	0.0514	0.0387	0.0572	0.0480
Western	Cold	0.0847	0.0838	0.0849	0.0890
	Comfort	0.0797	0.0748	0.0727	0.0766
	Tepidity	0.0347	0.0356	0.0369	0.0365

Through longitudinal comparison between regions, it can be seen that the comfortable climate fluctuations in the central and western regions decrease, while the fluctuations in the eastern region increase, indicating that the eastern region is the most livable in terms of temperature changes. The temperature changes in the central region are relatively stable, while the western region has more cold climates.

### 4.2 Characteristics of the depression of older adult people in rural areas

#### 4.2.1 Time trend of changes in psychological health of older adult people in rural areas by region

**Figure 3:**
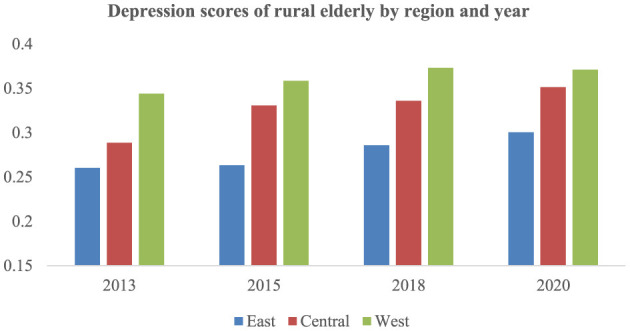
Depression scores of rural older adult in China by region and year.

This article presents the depression of older adult people in rural areas by region, as shown in Figure 3. Vertically, the depression scores of rural older adult in various regions have been fluctuating and slowly increasing. Horizontally, over the years, the average depression level of rural older adult has been the lowest in the east, followed by the middle, and the highest in the west.

#### 4.2.2 Classification of depression levels of rural older adult with different characteristics

From a micro perspective, this article compares middle-aged and older adult people in rural areas, and divides rural residents into older adult people with and without medical insurance, and older adult people with and without pension insurance. The time trend changes of depression scores for each group are shown in Table 3.

**Table 3:** Depression scores of rural residents in China by subgroup.

**Classification method**	**Characteristic group**	**2013**	**2015**	**2018**	**2020**
Comparison: middle-aged vs. older adult	Middle-aged group	0.2795	0.2734	0.3049	0.2910
	Older adult group	0.3003	0.3129	0.3308	0.3386
Comparison: with vs. without health insurance	Without health insurance	0.3341	0.3359	0.3318	—
	With health insurance	0.2988	0.3073	0.3308	0.3386
Comparison: with vs. without pension insurance	Without pension insurance	0.3150	0.3166	0.3370	0.3345
	With pension insurance	0.2986	0.2128	0.2458	0.3392

The 45–59 age group faces transitional-stage-specific depressive symptoms, balancing multiple pressures, including child-rearing and eldercare responsibilities while remaining active in the labor market. Based on considerations of intergenerational differences and policy guidance, this article compares the annual trends of depression scores between the older adult population (over 60 years old) and the middle-aged population (45–59 years old) based on age. The results are shown in Table 3. From this, it can be seen that firstly, the de-pression scores of the older adult population are generally higher than those of the middle-aged population. Secondly, the average depression score of the older adult population increases over time, while the depression score of the middle-aged population fluctuates over time. Middle-aged individuals can mitigate income loss from climatic shocks through labor mobility to climate-resilient non-agricultural sectors, whereas rural older adults, constrained by physical decline and reliance on climate-sensitive agriculture, face direct translation of economic vulnerability into psychological distress. This disparity explains the significantly higher cold-induced depression risk elevation in the older adult group (vs. middle-aged), highlighting systemic deficiencies in China's rural social security system for aging populations.

This article categorizes older adult people in rural areas based on whether they have medical insurance. The results are shown in Table 3. In 2020, due to the successful implementation of full coverage of rural medical insurance, there were no longer any data on the depression of older adult people in rural areas without medical insurance. It can be seen from this that in various years, the depression scores of rural older adult people with medical insurance are significantly lower than those without medical insurance, but the depression scores of rural older adult people with medical insurance are also in-creasing year by year. According to the classification of whether there is pension insurance, except for 2020, the depression scores of rural older adult people with pension insurance are significantly lower than those without pension insurance. However, the depression scores of rural older adult people with pension insurance only showed a significant decline in 2015, and gradually improved from 2015 to 2020, surpassing the level of 2013 in 2020. Overall, medical insurance and pension insurance do not have a sustained effect on reducing depression levels.

## 5 Results

### 5.1 Baseline regression on the impact of climate change on the depression of rural older adult

The baseline regression results for Model (1) are presented in Table 4. Column (1) shows that lower temperatures significantly increase depressive tendencies among rural older adults. Column (2) adds weather control variables, including precipitation, relative humidity, sunshine duration, and wind speed. Column (3) incorporates individual-level control variables, such as education level. Column (4) further controls for monthly time trends. The results from left to right indicate that an average monthly temperature below 5°C significantly increases depressive symptoms among rural older adults, suggesting that low temperatures exacerbate depression issues. Temperatures in the 5–10°C and 10–15°C ranges also raise depressive scores, though the effects are smaller compared to temperatures below 5°C. Column (4), which includes the most stringent controls, is treated as the baseline regression result. On the other hand, high temperatures (≥30°C) also positively affect CES-D scores, though the results are not statistically significant.

**Table 4:** Impact of climate change on the depression of rural older adults.

**Variable**	**CES-D (1)**	**CES-D (2)**	**CES-D (3)**	**CES-D (4)**
Monthly average temp. <5°C	1.509^*^	1.509^*^	1.835^**^	1.834^**^
	(0.9145)	(0.9142)	(0.7664)	(0.7661)
Monthly average temp. [5°C−10°C)	1.080^*^	1.343^**^	0.965^**^	0.965^**^
	(0.4589)	(0.5491)	(0.4441)	(0.4445)
Monthly average temp. [10°C−15°C)	0.560^**^	0.457^*^	0.337^**^	0.337^**^
	(0.1925)	(0.1925)	(0.1636)	(0.1637)
Monthly average temp. [15°C−20°C)	0.0634	0.0634	−0.0145	−0.0145
	(0.0772)	(0.0772)	(0.0729)	(0.00729)
Monthly average temp. [25°C−30°C)	−0.0323	−0.0323	−0.0202	−0.0201
	(0.0571)	(0.0571)	(0.0596)	(0.0604)
Monthly average temp. ≥30°C	0.0197	0.0701	0.0463	0.0048
	(0.0853)	(0.1318)	(0.1236)	(0.0124)
Individual fixed effects	Yes	Yes	Yes	Yes
City fixed effects	Yes	Yes	Yes	Yes
Year fixed effects	Yes	Yes	Yes	Yes
Month fixed effects	Yes	Yes	Yes	Yes
Monthly time trend	No	No	No	Yes
Observations	19,036	19,036	18,668	18,668
R^2^	0.4312	0.4312	0.4294	0.4294

(1) All control variables were regressed out during data preprocessing. (2) Fixed effects are included as dummy variables; their coefficients are not reported. (3) The reference temperature interval is [20°C−25°C). (4) Heteroskedasticity-robust standard errors clustered at the city level are reported in parentheses. (5) ^*^, ^**^, and ^***^denote statistical significance at the 10%, 5%, and 1% levels, respectively. The same applies to subsequent tables.

In the baseline regression, we further analyze the impact of extreme temperature changes on specific depressive symptoms using the CES-D scale. Higher scores indicate more severe symptoms. The results, presented in Table 5, show that low temperatures significantly increase the probability of depressive symptoms among rural older adults.

**Table 5:** Impact of climate change on specific depressive symptoms.

**Variable**	**Felt depressed**	**Lost hope**	**Felt lonely**	**Could not continue life**	**Difficulty concentrating**
Monthly average temp. <5°C	0.497^***^	0.151	0.299^***^	0.134	0.2463^*^
	(0.139)	(0.1255)	(0.0875)	(0.1576)	(0.13000)
Monthly average temp. [5°C−10°C)	0.0381	0.0689^*^	0.256	0.185^*^	0.051
	(0.2263)	(0.0418)	(0.1294)	(0.1122)	(0.1749)
Monthly average temp. [10°C−15°C)	0.0521	0.140^***^	0.134^***^	0.0743^**^	0.0761
	(0.0316)	(0.0435)	(0.0324)	(0.0368)	(0.0474)
Monthly average temp. [15°C−20°C)	0.0337^*^	0.0493^**^	0.0365	0.0105	−0.024
	(0.0163)	(0.0205)	(0.0233)	(0.0148)	(0.0297)
Monthly average temp. [25°C−30°C)	−0.0211	0.0275	0.0013	−0.0038	−0.0225
	(0.0162)	(0.0212)	(0.0101)	(0.0112)	(0.0155)
Monthly average temp. ≥30°C	−0.0057	0.0394	−0.0202	0.0007	−0.0085
	(0.0227)	(0.0417)	(0.0184)	(0.0912)	(0.0298)
Individual fixed effects	Yes	Yes	Yes	Yes	Yes
City fixed effects	Yes	Yes	Yes	Yes	Yes
Year fixed effects	Yes	Yes	Yes	Yes	Yes
Month fixed effects	Yes	Yes	Yes	Yes	Yes
Monthly time trend	Yes	Yes	Yes	Yes	Yes
Observations	18,201	17,465	18,317	18,151	17,858
R^2^	0.2913	0.1852	0.316	0.2717	0.2031

(1) All control variables were regressed out during data preprocessing. (2) Fixed effects are included as dummy variables; their coefficients are not reported. (3) The reference temperature interval is [20°C−25°C). (4) Heteroskedasticity-robust standard errors clustered at the city level are reported in parentheses. (5) ^*^, ^**^, and ^***^ denote statistical significance at the 10%, 5%, and 1% levels, respectively. The same applies to subsequent tables.

### 5.2 Robustness checks on the impact of climate change on the depression of rural older adult

#### 5.2.1 Alternative temperature intervals

To address potential sensitivity to temperature interval width, we re-estimate Model (1) using alternative intervals: 4°C (<5°C, 5–9°C, 9–13°C, 13–17°C, 17–21°C, 21–24°C, 25–29°C, ≥29°C) and 6°C (<5°C, 5–11°C, 12–17°C, 18–24°C, 23–29°C, ≥29°C). The reference group for the former is (21–24°C), and the reference group for the latter is (18–24°C).The results, shown in Table 6, are consistent with the baseline regression, indicating that the findings are robust to changes in temperature interval width.

**Table 6:** Robustness checks: alternative temperature intervals.

**(1)**		**(2)**	
**Using 4**°**C as the interval variable**	**Variable**	**Using 6**°**C as the interval variable**	**Variable**
Monthly average temp. <5°C	1.3742^**^	Monthly average temp. <5°C	1.3465^*^
	(0.7011)		(0.7878)
Monthly average temp. [5°C−9°C)	0.2958	Monthly average temp. [5°C−11°C)	0.4330
	(0.4058)		(0.3530)
Monthly average temp. [9°C−13°C)	−0.0030	Monthly average temp. [11°C−17°C)	−0.1267
	(0.2735)		(0.1899)
Monthly average temp. [13°C−17°C)	−0.0877	Monthly average temp. [23°C−29°C)	0.0111
	(0.2049)		(0.0821)
Monthly average temp. [17°C−21°C)	0.05415	Monthly average temp. ≥29°C	0.0328
	(0.1074)		(0.0572)
Monthly average temp. [25°C−29°C)	0.0489		
	(0.0863)		
Monthly average temp. ≥29°C	0.03647		
	(0.0572)		
Individual fixed effects	Yes		Yes
City fixed effects	Yes		Yes
Year fixed effects	Yes		Yes
Month fixed effects	Yes		Yes
Monthly time trend	Yes		Yes
Observations	18,668		18,668
R^2^	0.4291		0.4293

(1) All control variables were regressed out during data preprocessing. (2) Fixed effects are included as dummy variables; their coefficients are not reported. (3) The reference temperature interval is [20°C−25°C). (4) Heteroskedasticity-robust standard errors clustered at the city level are reported in parentheses. (5) ^*^, ^**^, and ^***^denote statistical significance at the 10%, 5%, and 1% levels, respectively. The same applies to subsequent tables.

#### 5.2.2 Alternative explained variable

The CHARLS survey includes a subjective assessment of recent life satisfaction. We construct a binary variable indicating depression (1 if CES-D score > 5, 0 otherwise) and estimate its relationship with climate change using a linear probability model. The results, shown in Column (1) of Table 7, indicate that a monthly average temperature below 5°C increases the probability of depression by 5.68%.

**Table 7:** Robustness checks: excluding the impact of COVID-19.

**Variable**	**Alternative DV**	**Placebo test**	**Excluding COVID-19**
Monthly average temp. <5°C	0.568^*^	0.1651	2.507^***^
	(0.3254)	(0.8287)	(0.4027)
Monthly average temp. [5°C−10°C)	0.118	−0.4625	0.760
	(0.0907)	(0.5684)	(0.4663)
Monthly average temp. [10°C−15°C)	−0.0161	−0.9494	0.202
	(0.0569)	(0.3253)	(0.1744)
Monthly average temp. [15°C−20°C)	−0.0008	−1.2579.	−0.0404
	(0.0157)	(0.2931)	(0.1032)
Monthly average temp. [25°C−30°C)	0.0008	−1.6825	0.0289
	(0.0099)	(0.3174)	(0.057)
Monthly average temp. ≥30°C	0.0129	−1.4460	0.0550
	(0.0198)	(0.3277)	(0.1212)
Individual fixed effects	Yes	Yes	Yes
City fixed effects	Yes	Yes	Yes
Year fixed effects	Yes	Yes	Yes
Month fixed effects	Yes	Yes	Yes
Monthly time trend	Yes	Yes	Yes
Observations	18,668	18,668	12,417
R^2^	0.3094	0.4292	0.4292

(1) All control variables were regressed out during data preprocessing. (2) Fixed effects are included as dummy variables; their coefficients are not reported. (3) The reference temperature interval is [20°C−25°C). (4) Heteroskedasticity-robust standard errors clustered at the city level are reported in parentheses. (5) ^*^, ^**^, and ^***^denote statistical significance at the 10%, 5%, and 1% levels, respectively. The same applies to subsequent tables.

#### 5.2.3 Placebo test

We conduct a placebo test using temperature variables from the month following the survey. The results, shown in Column (2) of Table 7, indicate no significant effects, supporting the robustness of the baseline findings.

#### 5.2.4 Excluding the impact of COVID-19

We further exclude the potential impact of the COVID-19 pandemic on depression. The results, shown in Column (3) of Table 7, confirm that climate change continues to significantly affect the depression of rural older adult.

### 5.3 Heterogeneity analysis

#### 5.3.1 Gender heterogeneity

This study first examines the heterogeneous effects of climate change on the depression of rural older adult residents by gender. The estimation results are presented in Columns (1) and (2) of Table 8. Low temperatures have a significant negative impact on depression of both males and females, but females are more sensitive to low temperatures.

**Table 8:** Heterogeneity by gender and educational level.

**Variable**	**Female**	**Male**	**High education**	**Low education**
Monthly average temp. <5°C	1.173^*^	1.144^*^	1.144^*^	1.834^**^
	(0.9145)	(0.6769)	(0.7664)	(0.7661)
Monthly average temp. [5°C−10°C)	1.425^**^	0.291	0.965^**^	0.965^**^
	(0.6813)	(0.4769)	(0.4441)	(0.4445)
Monthly average temp. [10°C−15°C)	0.513	0.0376	0.337^**^	0.337^**^
	(0.4093)	(0.2931)	(0.1636)	(0.1637)
Monthly average temp. [15°C−20°C)	0.0657	0.122	−0.0145	−0.0145
	(0.1308)	(0.1282)	(0.0729)	(0.00729)
Monthly average temp. [25°C−30°C)	−0.109	0.0399^*^	−0.0202	−0.0201
	(0.0674)	(0.097)	(0.0596)	(0.0604)
Monthly average temp. ≥30°C	−0.218	0.309	0.0589	0.0590
	(0.1508)	(0.1709)	(0.091)	(0.0913)
Individual fixed effects	Yes	Yes	Yes	Yes
City fixed effects	Yes	Yes	Yes	Yes
Year fixed effects	Yes	Yes	Yes	Yes
Month fixed effects	Yes	Yes	Yes	Yes
Monthly time trend	Yes	Yes	Yes	Yes
Observations	9,449	9,195	18,668	18,668
R^2^	0.4702	0.4916	0.4916	0.4916

(1) All control variables were regressed out during data preprocessing. (2) Fixed effects are included as dummy variables; their coefficients are not reported. (3) The reference temperature interval is [20°C−25°C). (4) Heteroskedasticity-robust standard errors clustered at the city level are reported in parentheses. (5) ^*^, ^**^, and ^***^denote statistical significance at the 10%, 5%, and 1% levels, respectively. The same applies to subsequent tables.

#### 5.3.2 Educational level heterogeneity

In this study, a high educational level is defined as having completed 9 years of compulsory education, while a low educational level is defined as not having completed 9 years of compulsory education. The regression results, as shown in Columns (3) and (4) of Table 8, indicate that under extreme low temperatures (monthly average temperature below 5°C), the score increase for the low education group is significantly higher than that for the high education group.

#### 5.3.3 Widowhood status

Numerous studies have shown that widowhood has various negative effects on the depression of the older adult, including reduced subjective wellbeing, increased loneliness, and depression (58). As shown in Column (1) of Table 9, the depression of rural older adult who have lost their spouses is more significantly affected by climate change.

**Table 9:** Heterogeneity by widowhood and occupational type.

**Variable**	**Widowhood**	**Agricultural Activity**
Monthly average temp. <5°C	0.129^*^	2.153^***^
	(0.0749)	(0.9491)
Monthly average temp. [5°C−10°C)	−0.161	1.214^*^
	(0.6205)	(0.5477)
Monthly average temp. [10°C−15°C)	−0.162^*^	0.555
	(0.2418)	(0.3612)
Monthly average temp. [15°C−20°C)	−0.126	0.0254
	(0.1772)	(0.086)
Monthly average temp. [25°C−30°C)	0.133	−0.0087
	(0.0995)	(0.0629)
Monthly average temp. ≥30°C	0.465^*^	0.0578
	(0.2129)	(0.0933)
Individual fixed effects	Yes	Yes
City fixed effects	Yes	Yes
Year fixed effects	Yes	Yes
Month fixed effects	Yes	Yes
Monthly time trend	Yes	Yes
Observations	3,042	17,125
R^2^	0.3987	0.4318

(1) All control variables were regressed out during data preprocessing. (2) Fixed effects are included as dummy variables; their coefficients are not reported. (3) The reference temperature interval is [20°C−25°C). (4) Heteroskedasticity-robust standard errors clustered at the city level are reported in parentheses. (5) ^*^, ^**^, and ^***^ denote statistical significance at the 10%, 5%, and 1% levels, respectively. The same applies to subsequent tables.

#### 5.3.4 Engagement in agricultural activities

Agricultural workers are often more dependent on climatic conditions, and extreme weather events can directly affect their productivity, thereby increasing psychological stress and anxiety. This study further distinguishes respondents engaged in agricultural activities. The regression results, as shown in Column (2) of Table 9, indicate that low temperatures significantly increase the CES-D scores of rural older adult engaged in agricultural activities compared to comfortable temperatures.

### 5.4 Mechanism testing

#### 5.4.1 Physical health

This study tests whether climate change worsens depression by harming physical health, with a specific focus on cardiopulmonary conditions. Chronic respiratory diseases—such as bronchial asthma, chronic obstructive pulmonary disease (COPD), chronic bronchitis, allergic rhinitis, and repeated respiratory tract infections (RRTL) in infants—serve as a critical pathway for this linkage, as they are known to exacerbate during winter months due to increased respiratory viral infections and exposure to low temperatures (59). To operationalize physical health status, this research leverages the CHARLS questionnaire to construct a binary variable (PH) reflecting cardiopulmonary health: respondents reporting no chest pain while climbing stairs (or uphill), the value is 1; otherwise, it is 0. This metric allows the study to quantify how climate-induced physical health deterioration mediates depression outcomes.

The estimation results of the mediation effect are presented in Columns (1) and (2) of Table 10. Column (1) tests Equation 3, and Column (2) tests Equation 4. The regression results show that **γ** is significant, and β^*^ρ and **γ** have the same sign, with an absolute value < **α** (1.834), indicating that physical health is a mediating variable through which climate change affects the depression of rural older adult. The empirical findings reveal that when the monthly average temperature is below 5°C, the coefficient is significantly negative, indicating that low temperatures increase the probability of cardiopulmonary discomfort, further leading to the deterioration of depression. Additionally, monthly average temperatures between 10°C and 15°C also increase the probability of physical discomfort, affecting the depression of rural residents. The coefficient in Column (2) (PH) is significantly negative, further indicating that good physical health effectively reduces the CES-D scores of rural older adult, improving their depression.

**Table 10:** Mechanism test (1).

**Variable**	**Physical health**	**Depression**
Monthly average temp. <5°C	−0.122^*^	1.8145^***^
	(0.073)	(0.7712)
Monthly average temp. [5°C−10°C)	−0.0823	0.9698^**^
	(0.1551)	(0.4472)
Monthly average temp. [10°C−15°C)	−0.223^**^	0.3496
	(0.0906)	(0.1673)
Monthly average temp. [15°C−20°C)	−0.0187	−0.0134
	(0.012)	(0.0730)
Monthly average temp. [25°C−30°C)	−0.00543	0.0198
	(0.0225)	(0.0602)
Monthly average temp. ≥30°C	−0.00476	0.0058
	(0.0299)	(0.0299)
PH		−0.0584^**^
		(0.0293)
Individual fixed effects	Yes	Yes
City fixed effects	Yes	Yes
Year fixed effects	Yes	Yes
Month fixed effects	Yes	Yes
Monthly time trend	Yes	Yes
Observations	18,668	18,668
R^2^	0.3214	0.4295

(1) All control variables were regressed out during data preprocessing. (2) Fixed effects are included as dummy variables; their coefficients are not reported. (3) The reference temperature interval is [20°C−25°C). (4) Heteroskedasticity-robust standard errors clustered at the city level are reported in parentheses. (5) ^*^, ^**^, and ^***^ denote statistical significance at the 10%, 5%, and 1% levels, respectively. The same applies to subsequent tables.

#### 5.4.2 Outdoor activity frequency

Studies have shown that more frequent physical exercise can alleviate depressive symptoms (60). This study uses the frequency of physical exercise among rural older adult as the main measure. Based on the CHARLS questionnaire, the number of outdoor activities in the past week is used as the explained variable to re-estimate Model (1). A binary variable (Outdoors) is constructed, where 1 indicates at least one outdoor activity in the past week, and 0 otherwise. A linear probability model is used to test whether climate change affects the frequency of outdoor activities on the extensive margin.

The estimation results of the mediation effect are presented in Columns (1) and (2) of Table 11. Column (1) tests Equation 3, and Column (2) tests Equation 4. The regression results show that **γ** is significant, and β^*^ρ and **γ** have the same sign, with an absolute value < **α** (1.834). The coefficient in Column (1) is significantly negative, indicating that climate change significantly affects the frequency of outdoor activities among rural older adult. The coefficient in Column (2) is significantly negative, indicating that reduced outdoor activities increase the CES-D scores of respondents.

**Table 11:** Mechanism test (2).

**Variable**	**Outdoor activity**	**Depression**
Monthly average temp. <5°C	−0.107^**^	1.7877^**^
	(0.0546)	(0.7688)
Monthly average temp. [5°C−10°C)	−0.0645	0.9194^**^
	(0.1596)	(0.4483)
Monthly average temp. [10°C−15°C)	−0.00877	0.2971^**^
	(0.056)	(0.269)
Monthly average temp. [15°C−20°C)	−0.0195	−0.0532
	(0.0227)	(0.0158)
Monthly average temp. [25°C−30°C)	0.00135	0.0162^*^
	(0.0194)	(0.0074)
Monthly average temp. ≥30°C	0.0058	0.0752^*^
	(0.0299)	(0.0891)
Outdoors		−0.0020^*^
		(0.0011)
Individual fixed effects	Yes	Yes
City fixed effects	Yes	Yes
Year fixed effects	Yes	Yes
Month fixed effects	Yes	Yes
Monthly time trend	Yes	Yes
Observations	18668	18668
R^2^	0.657	0.657

(1) All control variables were regressed out during data preprocessing. (2) Fixed effects are included as dummy variables; their coefficients are not reported. (3) The reference temperature interval is [20°C−25°C). (4) Heteroskedasticity-robust standard errors clustered at the city level are reported in parentheses. (5) ^*^, ^**^, and ^***^denote statistical significance at the 10%, 5%, and 1% levels, respectively. The same applies to subsequent tables.

#### 5.4.3 Cognitive ability

Deterioration in cognitive ability can also exacerbate depression issues. The cognitive ability section of the CHARLS questionnaire asked respondents about their mental status and situational memory ability, respectively. The mental status section, which is a reflection of cognitive integrity, consists of 10 questions, the details of which were obtained by testing the older adults' date cognition, numeracy, and drawing skills, respectively. In the date cognitive ability section, the interviewed older adult were required to answer the current year, month, day, season and week, and each correct answer counted for 1 point, totaling 5 points, and the higher the score, the stronger the date cognitive ability. The higher the score, the stronger the numerical ability; in the drawing ability section, the respondents were required to draw a picture of two five-pointed stars overlapping on a piece of paper presented by the tester, and one point was awarded for the successful drawing of the picture. The mental status score is the total of the above scores, out of 11 points, the higher the score, the better the mental status of the respondent, we will sum up the scores to get the total cognitive ability score of the respondent.

The estimation results of the mediation effect are presented in Columns (1) and (2) of Table 12. Column (1) tests Equation 3, and Column (2) tests Equation 4. The regression results show that **γ** is significant, and β^*^ρ and **γ** have the same sign, with an absolute value < **α** (1.834). The coefficient in Column (1) is significantly negative, indicating that climate change significantly affects the cognitive ability of rural older adult. The coefficient in Column (2) is significantly negative, indicating that a decline in cognitive ability increases the CES-D scores of respondents, worsening the depression of rural older adult.

**Table 12:** Mechanism test (3).

**Variable**	**Cognitive ability**	**Depression**	**Cognitive index**	**Depression**
Monthly average temp. <5°C	−0.0968^**^	1.7383^**^	0.0088^*^	1.7834^**^
	(0.0470)	(0.7934)	(0.0045)	(0.7934)
Monthly average temp. [5°C−10°C)	−0.592	0.9406^**^	−0.0538	0.9406^**^
	(0.9046)	(0.4556)	(0.0822)	(0.4556)
Monthly average temp. [10°C−15°C)	0.0359	0.3381^**^	0.00326	0.3381^**^
	(0.2959)	(0.1675)	(0.0269)	(0.1675)
Monthly average temp. [15°C−20°C)	−0.0255	−0.0155	−0.00232	−0.0155
	(0.1733)	(0.7293)	(0.0158)	(0.0729)
Monthly average temp. [25°C−30°C)	−0.178^*^	−0.0128	0.0162^**^	0.0128^*^
	(0.0815)	(0.0602)	(0.0074)	(0.0606)
Monthly average temp. ≥30°C	−0.359^*^	0.7376	0.0327^**^	0.0738
	(0.1619)	(0.0905)	(0.0147)	(0.0905)
Monthly average temp. <5°C		−0.0412^***^		
		(0.0063)		
Cogindex				0.4531^***^
				(0.0697)
Individual fixed effects	Yes	Yes	Yes	Yes
City fixed effects	Yes	Yes	Yes	Yes
Year fixed effects	Yes	Yes	Yes	Yes
Month fixed effects	Yes	Yes	Yes	Yes
Monthly time trend	Yes	Yes	Yes	Yes
Observations	18,668	18,668	18,668	18,668
R^2^	0.657	0.4295	0.657	0.657

Notes: (1) All control variables were regressed out during data preprocessing. (2) Fixed effects are included as dummy variables; their coefficients are not reported. (3) The reference temperature interval is [20°C−25°C). (4) Heteroskedasticity-robust standard errors clustered at the city level are reported in parentheses. (5) ^*^, ^**^, and ^***^denote statistical significance at the 10%, 5%, and 1% levels, respectively. The same applies to subsequent tables.

This study further constructs an Unhealthy Cognitive Ability Index (cogindex) to examine the proportion of individuals exhibiting psychologically unhealthy indicators, as shown in Equation 5:


(5) $$\begin{array}{l} CogIndex_{i}=\frac{\sum_{j=1}^{N_{j}}D_{ij}}{N} \end{array}$$

Where **N** is the total score of the cognitive health index variables (in this study, **N** = 11), and **D**_**ij**_ represents the value of the **j**-th depression variable for the i-th respondent. **D**_**ij**_ =1 indicates a health risk for the **j**-th depression variable, and **D**_**ij**_ =0 indicates a healthy state for the **j**-th depression variable.

The estimation results of the mediation effect are presented in Columns (3) and (4) of Table 12. Column (3) tests Equation 3, and Column (4) tests Equation 4. The regression results show that **γ** is significant, and β^*^ρ and **γ** have the same sign, with an absolute value < **α** (1.834). The coefficient in Column (3) is significantly positive, indicating that climate change increases the unhealthy cognitive index, worsening the cognitive ability of the older adult. The coefficient in Column (4) (Cogindex) is significantly positive, indicating that a higher unhealthy cognitive index leads to higher CES-D scores, worsening the depression of rural older adult. The results further confirm that monthly average temperatures below 5°C worsen the cognitive ability of respondents, and high temperatures also reduce cognitive ability.

#### 5.4.4 Cognitive ability

This study tests whether low temperatures affect the nighttime sleep duration of respondents. Adequate sleep duration has been proven to be closely related to depression (61, 62). Based on the CHARLS questionnaire, a sleep quality index (sleep) is constructed, where 1 indicates sleep duration between 6 and 9 h, and 0 otherwise. A binary variable (sleep) is used as the core explained variable, and a linear probability model is used to test the impact of high temperatures on sleep deprivation.

The estimation results of the mediation effect are presented in Columns (1) and (2) of Table 13. Column (1) tests Equation 3, and Column (2) tests Equation 4. The regression results show that **γ** is significant, and β^*^ρ and **γ** have the same sign, with an absolute value < **α** (1.834). The coefficient in Column (1) is significantly negative, indicating that compared to comfortable temperatures, monthly average temperatures below 5°C reduce the nighttime sleep quality of the older adult. The coefficient in Column (2) (sleep) is significantly positive, indicating that improved sleep quality effectively reduces CES-D scores, enhancing the depression of rural older adult. Therefore, residents experiencing low temperatures should be reminded to maintain adequate sleep, which helps mitigate the negative effects of low temperatures on depression. Additionally, the empirical analysis does not find significant effects of other temperature intervals on sleep duration or sleep deprivation.

**Table 13:** Mechanism test (4).

**Variable**	**Sleep quality**	**Depression**
Monthly average temp. <5°C	−0.153^*^	1.3768^**^
	(0.7312)	(0.7641)
Monthly average temp. [5°C−10°C)	−0.464	0.9630^**^
	(0.711)	(0.4458)
Monthly average temp. [10°C−15°C)	0.296	0.3363^**^
	(0.2187)	(0.1626)
Monthly average temp. [15°C−20°C)	−0.0842	−0.0151
	(0.0929)	(0.0729)
Monthly average temp. [25°C−30°C)	0.00956	−0.0201
	(0.0708)	(0.0604)
Monthly average temp. ≥30°C	0.0436	0.0591
	(0.1185)	(0.0913)
Sleep		−0.0309^*^
		(0.0155)
Individual fixed effects	Yes	Yes
City fixed effects	Yes	Yes
Year fixed effects	Yes	Yes
Month fixed effects	Yes	Yes
Monthly time trend	Yes	Yes
Observations	18,668	18,668
R^2^	0.1044	0.1021

(1) All control variables were regressed out during data preprocessing. (2) Fixed effects are included as dummy variables; their coefficients are not reported. (3) The reference temperature interval is [20°C−25°C). (4) Heteroskedasticity-robust standard errors clustered at the city level are reported in parentheses. (5) ^*^, ^**^, and ^***^denote statistical significance at the 10%, 5%, and 1% levels, respectively. The same applies to subsequent tables.

## 6 Discussion

This study deepens our understanding of climate-depression dynamics by systematically examining how temperature extremes (particularly chronic cold exposure) interact with socioeconomic vulnerability to influence psychological outcomes in a rural Chinese older adult population. Findings reveal three key aspects that reshape the current conceptual framework of environmental gerontology, while highlighting specific environmental pathways that require urgent policy attention.

First, this paper finds that cold is a major climate stressor for rural aging. In contrast to urban-centered studies that emphasize heat waves, this study finds that extreme cold is the primary climate risk factor for depression deterioration in rural areas-a finding with far-reaching implications for climate adaptation strategies. Rural older people face multiple vulnerabilities, such as agricultural livelihoods that exacerbate sensitivity to temperature fluctuations, and inadequate infrastructure that exacerbates exposure to cold. Observed gender differences suggest that females are more vulnerable, consistent with women's caregiving roles, which limit mobility and access to thermal protection resources. Cold-weather adaptation strategies should include subsidized home insulation programs and community heating centers targeting vulnerable subgroups such as widowed women and agricultural workers. Similarly, those engaged in agriculture were more affected, highlighting how climate risk can permeate occupational dimensions of depression, a pathway that has not been sufficiently explored in the existing literature. These findings suggest the need to reorient climate health policies to address cold weather resilience in rural areas, particularly in developing countries where older populations often live in thermally inefficient housing.

Second, the differential impacts observed across educational attainment, marital status and occupational groups reveal how climate risks interact with pre-existing social inequalities. Widowhood becomes a key vulnerability amplifier, possibly through a dual pathway: the loss of spousal support reduces the ability to implement adaptation measures, while grief exacerbates climate-related stress. Sensitivity was higher among subgroups with low levels of education, suggesting that limited health knowledge hinders awareness of cold weather risks and adoption of protective behaviors. These cross-cutting effects challenge homogenized approaches to climate adaptation, emphasizing the need for targeted interventions for overlapping marginalization. The mediating effect of social isolation—weather-induced mobility restrictions—further illustrates how climate change erodes community cohesion, transforming environmental stress into a chronic psychosocial burden.

Third, this study identified biopsychosocial pathways and systemic feedback loops through mediating effects testing. The identified mediating mechanisms-decreased physical health, limited outdoor activity, cognitive impairment, and sleep disruption-together depict a cascading model of climate-psychopathology. Cold-induced stress on the circulatory system may directly exacerbate depressive symptoms via neurovascular pathways while indirectly exacerbating psychological stress through functional limitations. Studies have found that physical health level decreases when the average monthly temperature drops below 5°C. Decreased physical health level was identified as a key factor in exacerbating depressive symptoms. Increased physical health level resulted in lower CES-D scores, which is a measure of depressive symptoms. This suggests that increasing physical health level has a direct and positive impact on depression. The findings emphasize that low temperature is an external condition that hinders physical health and indirectly affects depression. Although depression does not seem to be the main cause of physical ill-health among rural older adults in this context, a decrease in physical health level plays a central role in worsening depressive symptoms. The same applies to outdoor activity frequency, cognitive ability and sleep duration and quality etc.

Finally, the present study still has some limitations and is subject to improvement in future directions. While this study advances understanding of climate-depression linkages in rural China, several limitations warrant consideration. First, the reliance on depressive symptoms as the primary depression indicator limits exploration of broader psychological impacts like eco-anxiety or trauma. Future studies could employ mixed-method designs to capture nuanced emotional responses. Second, the focus on temperature variations does not fully account for compounding stressors like extreme precipitation or air quality changes, which may interact with cold exposure. Third, while the study identifies mediating pathways, unmeasured factors such as cultural attitudes toward depression or informal caregiving roles may influence outcomes. Longitudinal research tracking adaptive behaviors and policy interventions could clarify resilience-building strategies.

## 7 Conclusions

This study provides robust empirical evidence on the multifaceted impacts of climate change, particularly extreme low temperatures, on the depression of China's rural older adult population. Climate change has a significant negative impact on depression of rural older adult in China. Specifically, extreme low temperatures increase the tendency for depression among rural older adult, harming their depression. Extreme high temperatures also have a negative impact, but it is not statistically significant. Our findings reveal three key mechanisms that existing literature has not fully addressed: (1) The primacy of low-temperature effects over heat impacts in rural contexts contrasts with urban-focused studies emphasizing heatwaves, highlighting unique vulnerability profiles in agricultural communities. (2) The identification of agricultural engagement as an amplifier of climate-depression linkages extends previous economic vulnerability frameworks by demonstrating occupation-specific psychopathological pathways. (3) The quantification of cognitive decline as a critical mediator provides novel insights into neuropsychological pathways beyond the established physical health mechanisms. Three policy priorities emerge from these findings: (1) Climate-Resilient Healthcare Infrastructure: Develop mobile depression clinics equipped with cold-weather operation capabilities, prioritizing townships with >20% older adult populations. Integrate depression screening into existing rural primary care systems during winter months. (2) Social Protection Reinvention: Expand the rural pension system to include climate-adjusted payments during extreme cold periods. Pilot agricultural insurance products that compensate for depression service access during climate shocks. (3) Community-Based Adaptation: Implement “Warm Hub” programs in village committees, providing heated spaces for social interaction and cognitive stimulation during cold spells. Train farmer cooperatives as depression first responders through WHO's mhGAP program. Future research should investigate longitudinal climate exposure effects using decade-scale data and explore cultural protective factors through mixed-methods approaches. This research bridges critical gaps in environmental gerontology by providing the first nationwide analysis of climate-psychopathology mechanisms in rural China, offering a model for Global South populations facing similar demographic and climatic challenges.

## Data Availability

The original contributions presented in the study are included in the article/supplementary material, further inquiries can be directed to the corresponding author/s.
